# What Is New with Total Pancreatectomy and Autologous Islet Cell Transplantation? Review of Current Progress in the Field

**DOI:** 10.3390/jcm10102123

**Published:** 2021-05-14

**Authors:** Xavier L. Baldwin, Brittney M. Williams, Beth Schrope, Chirag S. Desai

**Affiliations:** 1 Department of Surgery, University of North Carolina in Chapel Hill, Chapel Hill, NC 27599, USA; xavier.baldwin@unchealth.unc.edu (X.L.B.); brittney.williams@unchealth.unc.edu (B.M.W.); 2 Department of Surgery, NewYork-Presbyterian/Columbia University Irving Medical Center, New York, NY 10032, USA; bs170@cumc.columbia.edu

**Keywords:** pancreatitis, chronic pancreatitis, total pancreatectomy, autologous islet cell transplantation, autoislet transplant

## Abstract

Patients with chronic pancreatitis have benefited from total pancreatectomy and autologous islet cell transplantation (TPAIT) since the 1970s. Over the past few decades, improvements have been made in surgical technique and perioperative management that have led to improved success of islet cell function, insulin independence and patient survival. This article focuses on recent updates and advances for the TPAIT procedure that continue to expand and innovate the impact on patients with debilitating disease.

## 1. Introduction

Early efforts at xenotransplantation and allotransplantation of islet cells in the late 19th and early 20th century cultivated the future forms of pancreatic surgery for patients with diabetes mellitus (DM) related complications and chronic pancreatitis [1,2]. David Sutherland performed the first human total pancreatectomy with autologous islet cell transplantation (TPAIT) at the University of Minnesota in 1977 for a patient with severe pancreatitis [3]. Although outcomes were initially poor, advancement in the field eventually enabled more sustained graft function and insulin independence reported after TPAIT as long as ten years following the initial procedure [4]. According to the Collaborative Islet Transplant Registry (CITR), a total of 819 auto-islet recipients have been reported to CITR between the years 1999 and 2015 with North America (11 of 23 sites), Europe (4 sites) and Australia contributing 754, 63 and 2, respectively [5]. Patients with chronic pancreatitis have benefited greatly from the innovation and evolution of pancreas surgery. Various surgical procedures have been created to combat morbidity associated with pancreatitis including operations that resect diseased portions of the pancreas and/or drain associated dilated ducts. Though not a routinely performed operation, TPAIT has been increasingly utilized for the treatment of patients with chronic pancreatitis in the last several years.

TPAIT results have shown that greater than 80% of patients are relieved of pain and eliminate the potential of future pancreatic malignancy due to pancreatectomy component. It also helps prevent brittle diabetes to an extent of insulin independence due to AIT factor [6]. The TPAIT procedure is complex and success is dependent on various factors starting from selection of patients, appropriate indications, surgical procedure execution, isolation and transfusion of cells, perioperative management and engraftment of cells in recipient sites. There has been progress and efforts in each phase of the surgical procedure. The purpose of this review is to highlight and update our understanding of contemporary practice in the field of TPAIT. We will describe recent advances in the clinical indications, preoperative evaluation, surgical techniques, islet isolation, alternative sites for implantation employed in the clinical setting and advances in management along with areas of future study. This update is focused only on the autologous islet cell transplant field.

## 2. Advances in Indications

Chronic pancreatitis and recurrent acute pancreatitis have served exclusively as the leading indications for TPAIT since its inception [7]. Patients depending on analgesics (mainly narcotic pain medications) requiring repeated hospital admissions and with well-preserved glycemic control are generally offered this therapy. Although additional indications are still controversial, exploring the expansion of this list has the potential to benefit a variety of patients. Investigations have taken place assessing the possibility of TPAIT in patients with diabetes, benign neoplasms, neoplasms with low malignant potential, patients where the risk of inadequate anastomosis and pancreatic fistulas are high and in rare pancreatic disorders such as pancreatic cystosis in patients with cystic fibrosis [8,9,10,11].

Traditionally, only patients with normal glycemic control were offered this procedure. When evaluating outcomes, understandable importance was previously on insulin independence, pain relief and improving quality of life, mostly in that order. Gradually, emphasis shifted from insulin independence to better control of DM and avoiding the subsequent brittleness [12]. Recently, there have been reports of this procedure offered to current diabetic patients showing advantages in management [13].

Balzano et al. published data from patients between 2008 and 2015 who underwent islet autologous cell transplantation (IAT) for benign and borderline neoplasms. Out of the 58 patients who underwent IAT, 31 had malignant tumors and 23 had pancreatic neoplasms with low or no malignant potential [10]. Overall, the average length of hospital stay was 12 days and the postoperative complication rate was approximately 65% with most complications categorized as minor. Although extent of pancreatectomy was an independent predictor of islet function, there was no difference in metabolic outcome (i.e., graft function, glycogenic control, insulin independence) with comparison of benign and neoplastic conditions. Greater than 90% of patients exhibited either insulin independence or partial graft function at 6 months after TPAIT. All patients with neoplasms of no or low malignant potential were disease free at the conclusion of the study. Relapse was observed in six (19.3%) patients of the malignant cohort who were initially disease-free following surgery [10]. Four patients developed ex novo liver metastases and two demonstrated metastatic disease progression. This is the only published series on malignancy; therefore, data are not sufficient to accept this as a practice. The recurrence of malignancy is certainly an argument against this if the comparative outcomes without AIT are not evaluated in detail. TPAIT indications for malignant cancers is still severely challenging; however, the safety and efficacy demonstrated by Balzano et al. with 14 neuroendocrine tumors and three other benign neoplasms provide the initial feasibility data needed to explore indications in benign tumors like solid-pseudopapillary lesions [10]. More studies and trials are required before conclusively advocating TPAIT for such lesions.

Cystic lesions of the pancreas with various pathology are increasingly diagnosed due to refinement on diagnostic modalities like CT scans and MRI. Considering variations in malignant potential and locations of cysts within pancreas, management can be challenging especially when distribution is diffuse throughout the pancreas. Intraductal papillary mucinous neoplasm (IPMN) and pancreatic cystosis have received interests for IAT given the often-diffuse extent of disease. The study mentioned above included 14 patients with cystic neoplasms with IPMN evident in 5 of these patients. There was no difference in postoperative outcomes, insulin independence, or graft function and survival when compared to other disease. A rare disease, but not uncommon, there is a lack of data describing the standard treatment for pancreatic cystosis in patient with cystic fibrosis. Systematic reviews show treatment of diffuse disease with pancreatectomy are comparable [14]. We reported the first successful case of islet cell auto transplantation for this indication with encouraging outcomes. While digesting the pancreas, it was noticed that exocrine tissue was minimal and walls of these cysts had islets with fibrous tissue. Upon digestion more than 5500 islet equivalent per kilogram of body weight was obtained with 75% purity and 90% cell viability. The patient has been insulin independent for almost 3 years up to follow up [11].

TPAIT is also considered for patients in situations where friable pancreatic tissue would compromise future anastomosis. Pancreaticoduodenectomies and operations for traumatic injury to the pancreas become highly morbid with the development of fistula or anastomotic leakage with rates reported between 2–15% [15]. Balzano et al. showed that patients with a potential high risk anastomosis benefit from TPAIT with comparable insulin independence and postoperative complications [9].

## 3. Advances in Evaluation

In regard to TPAIT, evaluation of endogenous insulin production is vital in order to predict future islet yield which reasonably corelate with insulin production post-operatively as well as good glycemic control without problems of hypoglycemia. Traditional tests include hemoglobin A1c (HbA_1c_), fasting serum glucose, fasting plasma C-peptide levels, fasting insulin levels and glucose and mixed meal tolerance tests [16]. The above measures have modestly predicted islet yield and future insulin independence. These tests perform suboptimal assessment of other cell functions within islets (other than beta cell function), which are responsible for preventing hypoglycemia related complications. In addition, if chronic pancreatitis patients are already diabetic then they do not accurately predict remnant function and insulin secretion. Arginine and glucagon stimulated C-peptide tests and continuous glucose monitoring have been shown to correlate with islet equivalents at operation and are used for the assessment of these patients [17,18].

C-peptide is part of the proinsulin molecule that is eventually cleaved prior to insulin secretion by β-cells. Its longer half-life than insulin affords a more stable test window of fluctuating β-cell response. Arginine is a positively charged molecule that leads to insulin/C-peptide secretion following depolarization of β-cells [19]. Recent studies have shown that preoperative c-peptide response to arginine correlates with islet mass during transplant [17]. McEachron et al. describes a 5 g intravenous injection of arginine followed by C-peptide measurements at 2, 3, 4, 5, 7 and 10 min time points [17]. In contrast to arginine, glucagon stimulates insulin/C-peptide secretion via glucagon and glucagon-like peptide 1 receptors on β-cells. Traditionally, 1 milligram of glucagon is given intravenously over 10 s. Subsequent C-peptide measurements are at 2, 4 and 6 min intervals.

Continuous glucose monitoring (CGM) could also serve as a preoperative indicator for islet yield. Technologic advances have allowed more practical and accessible subcutaneous devices to continually measure glucose levels. Given this recent feasibility, Beltran et al. completed a study that illustrated presurgical CGM markers that assess deviation from glucose homeostasis accurately predict islet yields [20]. This test shows promise in prediction of TPAIT outcomes as it also gives an idea about percentage of time patients have hypoglycemia over the examined period of a few days. The test is easy and less cumbersome amongst any other test applied for beta cell evaluation.

The presence of pre-diabetes defined by the American Diabetic Association (fasting plasma glucose between 100 and 125 mg/dl (5.6–6.9 mmol/L) or HbA1c between 5.7% and 6.4% without any pharmacological support), was correlated with a significantly lower chance for insulin independence at 1 year, 13% vs. 53% in patients with normal glucose control, which has major clinical implications. Information about HbA1c and fasting blood glucose are easily obtained to help in preoperative decisions and set up realistic expectations about glucose control postoperatively [13].

## 4. Advances in Technique

Over the last decade, certain subtleties in surgical technique have shown benefits in both patient outcome and islet yield. First, maintaining major vascular supply throughout dissection by dividing the splenic and gastroduodenal arteries last prevent ischemia and thus reduce islet loss. Second, removing the organ in total, i.e., not dividing it at the neck, has been shown to reduce organ inflammation and preserve islets [21]. Recently, there has been momentum gaining toward more minimally invasive TPAIT procedures. For various surgical operations, minimally invasive approaches improve cosmesis, length of hospital stay and decrease postoperative pain. Though extremely limited in application and restricted to case series, early experience in laparoscopic and robotic procedures have been demonstrated to be feasible and safe; however, better and larger data is required to confirm safety, efficacy and operative efficiency. Berger et al. analyzed a cohort of 42 patients who underwent hand assisted laparoscopic TPAIT vs. open TPAIT and found comparable rates of length of stay, postoperative narcotic use and quality of life [22]. The laparoscopic group did experience higher rates of bowel obstruction, bile and chyle leaks (52%) when compared to 14% of the open group [22]. Fan et al. also described a cohort of 20 patients who underwent total laparoscopic TPAIT [23]. Operative times were reported shorter than typical open procedures with average times of 493 min. Cases selected may have been biased towards recurrent acute pancreatitis pathology as opposed to the more difficult scarred pancreas. However, they did not perform vascular preservation to reduce warm ischemia time (WIT) and removed the pancreas in two parts. Many state that robotics have improved visualization and dexterity compared to laparoscopy and open procedures. Robotics may also help to improve the integrity of gastrointestinal reconstruction compared to laparoscopy and avoid unwanted complications. Although robotic TPAIT is not performed often, Galvani et al. reported on a series of 6 patients where one third of patients experienced postoperative morbidity [24]. There were longer operative times with an average total operative time of 712 +/− 74.1 min [24]. Surgeons abided by the critical issues of vascular preservation and resection of the entire pancreas [25]. An additional obstacle are the higher costs and steep learning curve associated with robotic surgery.

## 5. Advances in Isolation

The digestion of the pancreas is a critical step in determination of future islet yield and function. Briefly, the isolation procedure includes trimming the fat, blood vessels and connective tissues and washing the pancreas in a solution of antibiotics. The pancreatic duct is cannulated and after enzymatic infusion, the pancreas is distended and increased in volume by a substantial margin. The pancreas is then cut into small pieces (about 1–2 cm^3^) and the tissue transferred to the Ricordi chamber for digestion. If the total tissue volume is more than body weight × 0.25 mL/kg, purification is performed [26]. Once digestion is completed and the pancreatic islet cells isolated, several washes are performed. The final islet cell product is analyzed by gram stain for sterility, endotoxin and viability and mixed with albumin and heparin for infusion.

In contrast to the healthy pancreas found in deceased donors for allogeneic transplantation, pancreases removed from chronic pancreatitis patients are often fibrotic, calcified, or infiltrated with fat (often seen in cystic fibrosis patients). In addition, many of these patients have had prior surgery, either resections or drainage procedures, resulting in less parenchyma from which to extract islets. Due to these circumstances, adjustments in the isolation procedure in terms of collagenase enzyme dosing are made to obtain optimal mass and quality of transplantable islets. New developments show that several adjustments including weight-based dosing of collagenase enzymes, enzyme distension for prolonged periods, parenchymal injection and hand injection as opposed to pump injection may lead to increase islet yield [27].

## 6. Advances in Alternate Sites

Traditionally, in TPAIT islet cells have either been transplanted into the liver or rarely into the peritoneum. Transfusion of islet cell preparation directly into the portal venous circulation into the liver can be undertaken during open operation via the splenic vein stump/small venotomy in accessible extrahepatic portal vein or percutaneously via transhepatic portal vein infusion. In some patients, these routes are challenging (for example, due to prior thrombosis of these vessels or during transfusion when portal pressure gets very high); in those cases, cells are implanted in omental pouch or in peritoneal pockets. Over the past few decades, the islet community has realized that the liver is only the best amongst other sites but not a physiologically ideal site. Upon transplanting these islets into the liver, a nonspecific immune response—mediated predominantly by site-dependent innate inflammatory events—combined with pre-existing and transplant-induced cellular immune responses, contribute to relative hypoxia in portal venous circulation that plays a major role in islet loss [25,28,29,30]. With these challenges, it is imperative to develop adjuvant therapies such as delivery of trophic factors and alternative sites such as muscle or bone marrow that promote immune homeostasis and angiogenesis to improve the hepatic microenvironment for successful IAT [31].

In a mixed murine model and human study presented by Christoffersson et al., islets engrafted to the musculature exhibited earlier neovascularization compared to liver grafts [32]. In this study, the islets of three patients receiving TPAIT were successfully transplanted to the brachioradialis muscle and MRI was the technique of choice to accurately visualize the grafts. Fractional plasma volume was used to correlate with surrounding capillary density compared to adjacent muscle [32]. In addition to imaging techniques to characterize islets implanted to muscle, glucagon-like peptide 1 (GLP-1) has been used as a reliable beta-cell marker. Pattou et al. presented a patient that underwent extended pancreatectomy and autologous-islet transplantation with confirmation of functioning islets one year post transplant utilizing a radiolabeled GLP-1 analogue [33].

Similar to muscle engraftment, bone marrow can potentially serve as a site for islet transplantation avoiding the immunologic, anatomic and metabolic factors associated with liver implantation that leads to early graft loss. A study completed by Maffi et al. followed 4 patients who received a bone marrow islet infusion to the iliac crest. Follow up studies confirmed engraftment and function with circulating C-peptide levels and stable HgA_1c_ [34]. This study also confirmed no significant impact on endogenous production of peripheral blood cells with bone marrow infusion. These findings help establish the bone marrow as a potential future site for implantation.

## 7. Advances in Management

Patient management during the first few days following islet cell transplantation influence outcomes. Universal measures applied to all surgical patients to help minimize infections, hemorrhage and stress responses are observed during the perioperative period. In addition, specific efforts are made to rest the islet cells during initial engraftment with an intravenous insulin protocol along with anticoagulation to help prevent portal vein thrombosis and platelet aggregation [35]. Instant blood mediated inflammatory responses that happen involving coagulation and complement activation causes significant islet mass loss upon infusion into the portal vein [36]. This inflammatory response can lead to destruction and apoptosis contributing to islet damage prior to neovascularization. Szempruch et al. completed a systematic review recently examining the use of different anti-inflammatory agents in islet cell transplantation. Common medications observed in use currently include Etanercept (ETA) and Infliximab, tumor necrosis factor (TNF) blockers and Anakinra (ANA), an interleukin-1 receptor antagonist (IL-1RA) [37]. Infliximab is now rarely used and typical regimens involve several dosages of combination therapy (ANA + ETA). Although future studies are needed for standardization, there is a potential clinical benefit from the use of medications that limit the immune response. Alpha 1 anti-trypsin investigation led to its use to suppress macrophage activation promoting islet graft survival; however, widespread use beyond a single center has not been validated [38]. Similarly, there has been some research interest in mesenchymal stem cell co-transplantation with islet cells to improve engraftment [NCT02384018]. Desai et al. show high variability of practice but some generalizations regarding the concern for hypercoagulable status, use of unfractionated heparin intraoperatively and use of anticoagulation with low molecular weight heparin in the postoperative period. Practices of anticoagulation have been inconsistent and protocols will need to be unified [39].

Surveillance following TPAIT is instrumental in assessing islet graft status. Progressing islet amyloidosis after intraportal infusion has been suggested responsible for islet cell decline after transplant. However, this theory has been challenged recently based on clinical observations and pathological findings [40,41]. Insulin release stimulation assays allow accurate evaluation and monitoring of islet graft function following transplant, but these tests are logistically challenging given the often far proximity of patients to transplant centers and less reliable individual fasting values. The BETA-2 score which combines values of fasting blood glucose, c-peptide, HbA_1c_ and insulin requirements was developed for islet graft function assessment after allotransplantation and has recently been validated in the setting of autotransplantation. This serves as a reliable measurement for the monitoring of graft function in patients undergoing TPAIT [42].

In conclusion, clinical auto islet cell transplantation has made progress in the last decade. More centers are offering this surgery to patients with a variety of indications. Surgical techniques for minimizing incisions and islet damage are evolving and being standardized. Progress in isolation techniques have been very successful and investigation remains for potential alternate sites. Peri-operative management is focused on increasing islet engraftment with various strategies and future studies are optimistic for continued improved in long term graft survival.

## Data Availability

Not applicable.
